# Metabolomics Combined with Proteomics Provide a Novel Interpretation of the Changes in Flavonoid Glycosides during White Tea Processing

**DOI:** 10.3390/foods11091226

**Published:** 2022-04-24

**Authors:** Xuming Deng, Hu Shang, Jiajia Chen, Jun Wu, Tao Wang, Yiqing Wang, Chensong Zhu, Weijiang Sun

**Affiliations:** 1 College of Horticulture, Fujian Agriculture and Forestry University, 15 Shangxiadian Road, Fuzhou 350002, China; 15280766637@163.com (X.D.); huzi0538@163.com (H.S.); chenjiajia0593@163.com (J.C.); wuj081896@163.com (J.W.); wangtwtao0827@163.com (T.W.); wangty0912@163.com (Y.W.); 2 Fujian Xi Ming Tea Industry Co., Ltd., Fuding 355200, China; zhuchensong@ximingcha.com

**Keywords:** flavonoid glycosides, withering processing, white tea, WGCNA, oxidative stress

## Abstract

In this study, nonvolatile metabolomics and proteomics were applied to investigate the change mechanism of flavonoid glycoside compounds during withering processing of white tea. With the extension of withering time, the content of the main flavonoid glycoside compounds significantly decreased, and then the flavonoid aglycones and water-soluble saccharides contents increased. However, the change trends of these compounds were inconsistent with the expression pattern of related biosynthesis pathway proteins, indicating that the degradation of flavonoid glycosides might exist in the withering process of white tea. One co-expression network that was highly correlated with variations in the flavonoid glycosides’ component contents during the withering process was identified via WGCNA. Further analysis revealed that the degradation of flavonoid glycosides may be related to the antioxidant action of tea leaves undergoing the withering process. Our results provide a novel characterization of white tea taste formation during processing.

## 1. Introduction

Based on the different sensory, flavor, and processing procedures of tea products, tea can be classified into oolong tea, black tea, yellow tea, dark tea, white tea, or green tea. White tea (*Camellis sinensis* (L) O. Kuntze) has gradually attracted the attention of the public due to its special flavor, significant health care functions, and resistance to deterioration in storage [1,2,3]. There is a significant difference in nonvolatile components between white and other types of teas due to the differences in fresh tea sources and processing processes [4]. Compared with other teas, the processing process of white tea is the simplest. It simply needs to go through a long wilting and drying process without any need for roasting, mechanical injury, or microbial fermentation.

Remarkably, the prolonged withering process is thought to have a crucial role in the distinctive chemical makeup of white tea. This production process, similar to that of Chinese herbal medicines, gives white tea unique flavors, referring to the gradual transformation from bitterness and astringency to a slight sweetness and umami (a subtle, slightly sweet, refreshing taste) [5,6]. The flavonoid glycosides, a subcategory of the flavonoid chemical family and a third of all tea polyphenols, are also the important contributing components of this astringent flavor transformation process in white tea [7,8]. Additionally, the flavonoid glycosides, the main components of which include flavone glycosides, flavonol glycosides as well as their derivatives, mainly exist in the form of O-glycosides with a glycoside moiety at the C-3 position of aglycones [9]. In addition, due to their comparatively greater stabilities related to catechins, flavonoid glycosides are regarded as a set of interesting biomarkers for differentiating the origins or cultivars of teas [10].

In an earlier study examining the effect of tea processing on changes in flavonoid glycosides, Chen et al. concluded that during the processing of white tea, the levels of some compounds, such as apigenin-6–8-diglucoside, apigenin-6-glucoside-8arabinoside, luteolin-8-glucoside, apigenin-8-glucoside-6-arabinoside, and kaempferol-3,7-di-rhamnoside, showed an increasing trend [6]. Fang et al. found that the content of total flavonol glycosides exhibited decreasing trends throughout the withering stage of white tea, which contradicted the findings of Chen et al. [11]. Tan et al. also concluded that flavonol glycosides with the same aglycon showed comparable patterns during the fermentation of black tea. Furthermore, the abundances of kaempferol and quercetin glycosides declined within the first hour, after which they remained in stable states [12]. However, according to Dou et al., flavonol glycosides did not deteriorate significantly throughout the semi-fermentation phase of oolong tea, but 20% of total flavonol glycosides decomposed during the subsequent drying process. These debatable outcomes are linked to the absence of systematic studies on the mechanisms of flavonoid glycoside changes during the manufacture of different tea types [13].

In the prolonged withering process, the moisture content of harvested fresh leaves of tea plants is continuously reduced (from 70–78% to 20–30%), during which the leaves are under strong non-biological stress and aging is accelerated. At the same time, the leaf cells stay active throughout the initial stages of withering and then gradually disintegrate during the late stages, resulting in wide metabolic changes. Some studies have provided insights into the biochemical processes of flavonoid glycosides during this withering period. Most of the genes related to flavonoid biosynthesis were suppressed at the transcriptional level within 24 h of withering processing, suggesting that the reduction in flavonoid content may also correlate with a decrease in flavonoid biosynthesis [14]. Guo et al. demonstrated through in vitro tests that polyphenol oxidase (PPO) has higher catalytic activity for the conversion of flavonoid glycosides in tea compared to peroxidase POD and β-glucosidase, which may be due to the fact that the sugar moiety increased the docking affinity of flavonol glycosides for PPO [15]. However, in this fundamental molecular pathway, the production outcome of the changes in flavonoid glycosides has not been thoroughly studied in white tea.

The main goals of this study were to look at the dynamic changes in flavonoid glycosides throughout withering using a metabolomics method, and then, by integrating proteomics data, to investigate the molecular mechanism behind the modifications in flavonoid glycosides at the “protein–metabolite” level. This research will add to our knowledges of the mechanisms underlying the production of white tea flavor and quality control throughout the production procedure.

## 2. Materials and Methods

### 2.1. Reagents and Chemicals

Correspondingly, formic acid and methanol were acquired from TIC Corporation (Tokyo, Japan) and Merck Corporation (Darmstadt, Germany). Ultrapure deionized water was manufactured by a Milli-Q water refining system (Millipore, Billerica, MA, USA).

### 2.2. Tea Sample Processing

The tea cultivar of Fuding Dahao (*Camellis sinensis* (L) O. Kuntze) was selected for manufacturing white teas in this research. A total weight of 45 kg of tea leaves composed of one bud with two leaves were picked to process tea samples. The processing site was the ‘xiangheshan’ production base of the Pinpinxiang Tea Industry Co. Ltd., located in Fuding City, Fujian, China. After being transported to the production base, the fresh leaves were spread out in the withering room and divided into three parallel replicates, 15 kg each. The main processing parameters were as follows: fresh leaves were wilted at 25 °C for 8 h in the first stage, then at 30 °C for 16 h, and at 38 °C for 6 h in the last stage, with an air humidity of 60–80%, then the leaves were spread out into bamboo sieves with mesh to a thickness of 2–3 cm (approximately 1 kg per sieve). The withered leaves could not be randomly removed. Throughout the processing time, tea samples were taken uniformly from withered leaves at time points of 0 h, 12 h, and 30 h, and fixed with liquid nitrogen. Then, 100 g of tea material was sampled for each technical replication, repeated three times for a total of 300 g. Moreover, the leaves were sampled at different points throughout the withering area and pooled to create a composite sample. At the same time, the weight, moisture content, and temperature of the tea leaves at each time interval were monitored with an electronic scale (EKS-L221, Etekcity, Los Angeles, CA, USA), moisture analyzer (MA150C-000230V1, Sartorius; GER), and handheld infrared thermometer (62 MAX, Fluke; Everett, WA, USA), respectively. For proteomic studies, frozen tea leaves were kept at −80 °C (Table S1B). A fraction of frozen tea leaves was freeze-dried (FD5–10, SIM International; Los Angeles, CA, USA) at −55 °C for 36 h. For the metabonomic study, these freeze-dried tea leaves were kept at −20 °C (Table S1A).

### 2.3. Analysis of Total Flavonoids and Total Water-Soluble Saccharides

Spectrophotometric techniques were used to determine the chemical compositions of tea samples, such as total flavonoids and total sugars [16,17].

### 2.4. Widely Targeted Metabolomic Analysis

#### 2.4.1. Extraction and Processing of Samples

Using of a mixer mill (MM 400, Retsch) with a zirconia bead, the freeze-dried tea leaves were pulverized for 1.5 min at 30 Hz. Then, 100 mg of the resulting powder was measured and extracted overnight at 4 °C with 0.6 mL 70% aqueous methanol. The components were absorbed onto an SPE cartridge after centrifugation at 10,000× *g* for 10 min (CNWBOND Carbon-GCB SPE Cartridge, 250 mg, 3 mL; ANPEL, Shanghai, China, www.anpel.com.cn/cnw, accessed on 20 December 2020) and filtrated (SCAA-104, 0.22 μm pore size; ANPEL, Shanghai, China, http://www.anpel.com.cn/, accessed on 20 December 2020) before UPLC-MS/MS analysis.

#### 2.4.2. UPLC Conditions

The sample extracts were analyzed using an UPLC-ESI-MS/MS system (UPLC, Shim-pack UFLC SHIMADZU CBM30A system, www.shimadzu.com.cn/, accessed on 20 December 2020; MS, Applied Biosystems 4500 Q TRAP, www.appliedbiosystems.com.cn/, accessed on 20 December 2020). The analytical conditions consisted of a UPLC column, Waters ACQUITY UPLC HSS T3 C18 (1.8 µm, 2.1 mm × 100 mm); The mobile phase was made up of Solvent A, clean water having 0.04% acetic acid; and acetonitrile with 0.04% acetic acid, Solvent B. The specimen was analyzed using a gradient algorithm with 95% A and 5% B as the initial settings. Within 10 min, a linear gradient to 5 percent A, 95 percent B was designed, and the composition of 5% A, 95% B was maintained for 1 min. Following that, a 95% A, 5.0% B composition was altered in 0.10 min and held for 2.9 min. The temperature in the column oven was set to 40 degrees Celsius. The injection volume was set at 4 μL. The effluent was coupled to an ESI-triple quadrupole-linear ion trap (QTRAP)-MS as a substitute.

#### 2.4.3. ESI-Q TRAP-MS/MS

On a triple quadrupole-linear ion trap mass spectrometer (Q TRAP), API 4500 Q TRAP UPLC/MS/MS System, coupled with an ESI Turbo Ion-Spray interface, running in positive and negative ion modes and managed by operator 1.6.3 software, LIT and triple quadrupole (QQQ) scans were produced (AB Sciex). The ESI source operation characteristics were ion spray voltage (IS), 5500 V (positive ion mode)/−4500 V (negative ion mode); ion source, source temperature 550 °C; turbo spray; ion source gas I (GSI), gas II(GSII), curtain gas (CUR), set at 50, 60, and 30.0 psi, respectively; CAD (collision gas) was high. In QQQ and LIT modes, instrument tuning and mass calibration were done with 10 and 100 mol/L polypropylene glycol solutions, accordingly. MRM tests were used to obtain QQQ scans, with the collision gas (nitrogen) adjusted to 5 psi. Additional DP and CE tuning were performed for specific MRM transitions. According to the metabolites eluted at each phase, a specified set of MRM transitions was tracked.

#### 2.4.4. Differential Metabolites Selected

Variable importance in projection (VIP) ≥ 1 and absolute Log2FC (fold change) ≥ 1 were used to identify metabolites that were considerably regulated across groups. VIP numbers were extracted from the OPLS-DA results, which comprised integrated permutation plots and score plots, and were created using the MetaboAnalystR R package. Before OPLS-DA, the data were log-transformed (log2) and mean-centered. A randomization test (200 permutations) prevented a fitting problem.

### 2.5. Quantitative Proteomic Assessment Using Tandem Mass Tags (TMT)

#### 2.5.1. Protein Extraction and Peptide Digestion

Tea samples were digested with SDT (4% (*w*/*v*) SDS, 100 mM HCl/Tris pH 7.6, 0.1 M DTT) to extract proteins [18].

#### 2.5.2. High pH and TMT-Labeling Grading of Reversed-Phase Peptides

Using TMT reagents as per the manufacturer’s guidelines, each specimen had 100 g of peptide composition labeled (Thermo Scientific, Waltham, MA, USA). TMT-labeled digested specimens were fractionated into 15 fractions by means of a Pierce high pH reverse-phase fractionation kit (Thermo Scientific) and an increased acetonitrile step-gradient elution was performed as per the directions.

#### 2.5.3. LC-MS/MS Analysis

For 60 min, an Easy nLC linked to a Q Exactive mass spectrometer (Thermo Scientific, Waltham, MA, USA) was used to evaluate liquid chromatography–mass spectrometry (LC-MS/MS). The mass spectrometer was operated in positive ion mode. The MASCOT engine (Matrix Science, London, UK; version 2.2) incorporated in Proteome Discoverer 1.4 was used to search for tandem mass spectrometry (MS/MS) spectra.

#### 2.5.4. Identification and Quantitation of Proteins

The mass spectrometry data were saved in RAW format, and library identification and quantitative analysis were performed using the tools Mascot 2.2 and Proteome Discoverer 1.4. The following were the characteristics: the highest missed cleavages were two; TMT 6/10 plex (N-term), carbamidomethyl (C), and TMT 6/10 plex (P-term) were the three fixed alterations (K); the variable variations were TMT 6/10plex (Y) and oxidation (M); the peptide and fragments’ mass tolerances were 20 ppm and 0.1 Da, respectively; the protein ratio was derived using the median of the protein’s only distinct peptides; peptides and proteins had a 0.01 false discovery rate; the median protein ratio was used to balance all peptide ratios, and the median protein ratio must be 1, following normalization; for analysis of protein, the tea genome (*Camellia sinensis* var. assamica) database was employed [19], and the database structure for calculating FDR was a decoy. Fold-change values larger than ±1.2 and *p* < 0.05 (from Student’s *t*-test) were utilized to find differentially expressed proteins.

#### 2.5.5. Annotation and Enrichment Study of GO and KEGG Pathways

The Gene Ontology (GO) functional annotation of all proteins discovered in this experiment was conducted using Blast2Go (https://www.blast2go.com/, accessed on 20 December 2020)) [20] software. Fisher’s exact test technique was used to analyze GO functional enrichment of differential expression. The protein arrangements of differentially articulated proteins were blasted to extract their KEGG Orthologies from the online Kyoto Encyclopedia of Genes and Genomes (KEGG) database (http://geneontology.org/, accessed on 20 December 2020) (KOs). Following that, they were linked to KEGG routes. The pathways of corresponding KEGG were retrieved. Using Fisher’s exact test, KEGG routes enrichment study was undertaken by means of all measured protein descriptions as background datasets. The resulting *p*-values were then adjusted with the help of the Benjamini–Hochberg adjustment for multiple testing. *p*-value-based routes smaller than 0.05 were measured as significant.

### 2.6. Processing of Data

Principal component analysis (PCA) without supervision (basic element examination) was conducted using statistics function prcomp within R (www.r-project.org, accessed on 20 December 2020). Before unsupervised PCA, the data were adjusted by unit variance. Graph Prism 7.0 software (GraphPad, San Diego, CA, USA) was used to draw bar and line graphs. A co-expression controlling network was manufactured by WGCNA.

The WGCNA R program was used to conduct the weighted gene co-expression network analysis (WGCNA) (Langfelder and Horvath, 2008). The co-expression network was built using proteins with varying expression recognized by fold modified values larger than ±1.2 and *p* < 0.05 (from Mann–Whitney U-tests). A matrix of bilateral Pearson correlation coefficients (PCC) between all pairings of genes was constructed, as stated by Zhang et al. [21]. After that, the matrix was turned into a data structure by increasing the co-expression parameter (0.5 + 0.5 × PCC). The optimal soft threshold in this investigation was power β = 13, and the resulting adjacency matrix was utilized to determine the topological overlap (TO). The genes were hierarchically grouped based on TO resemblance, and the hierarchical clustering tree was cut using the dynamic hybrid tree cut technique. For module identification, the lowest module size was fixed to 30, while the lowest height for combining modules was fixed to 0.25. The first main component of the scaled gene expression profiles was used to summarize each component (module eigengene, ME). Depending on PCC assessment, the content values of the 26 main nonvolatile compounds were employed as phenotypic variables for module–trait correlations. Gene significance (GS) was utilized to connect characteristic data with specific gene expression data. Cytoscape (Version 3.9.1, Cytoscape Consortium, Bethesda, MA, USA) was used to view the module networks.

## 3. Results and Discussion

### 3.1. Modifications in Water Content and Overview of Nonvolatile Constituents (Flavonoids and Water-Soluble Saccharides) in Tea Leaves during Withering

**Figure 1:**
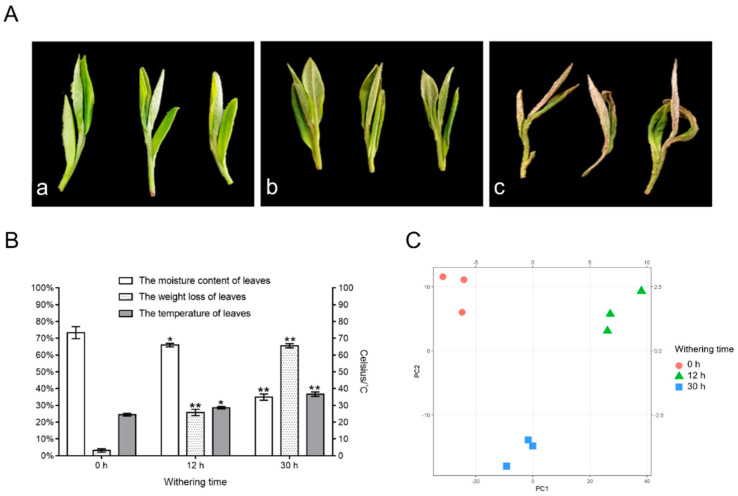
The tea leaf changes in water content, weight loss, temperature, and overview of nonvolatile compounds. (**A**) The phenotype of the picked tea leaves at 0 h (a), 12 h (b), and 30 h (c). (**B**) The moisture content, weight loss, and temperature of tea leaves following withering for different time periods. Data represent the mean value ± standard deviation (n = 3), * *p* < 0.05 compared with the sample of 0 h, ** *p* < 0.01 compared with the sample of 0 h. (**C**) Principal component analysis score plot of tea leaves with different withering times (0 h, 12 h, and 30 h).

The dehydration rate of leaves is directly connected to changes in the concentration of their flavor components, and withering plays a significant part in producing the taste of white tea [22]. To discover time-dependent variations in flavonoid glycosides, the water content and nonvolatile compounds (flavonoids and saccharides) stages were evaluated at 0 h, 12 h, and 30 h of withering. As presented in Figure 1A, with the lengthening of the withering period, the brilliance of the new leaves faded with time, as did their color intensity, and leaf borders contracted and withered. The leaf temperatures also increased gradually. At 0 h, 12 h and 30 h, the leaf temperatures were 24.5 ± 0.8 °C, 28.6 ± 0.72 °C, and 36.7 ± 1.32 °C, respectively (Figure 1B). Water content and weight loss determination demonstrated that the moisture content and weight loss of fresh leaves were estimated to be 73.32 ± 3.6% and 3.12 ± 1.01%, respectively (Figure 1B). After withering for 12 h, the moisture content and weight loss determination reached to 65.9 ± 1.06% and 25.74 ± 1.9%, correspondingly. After 30 h, the moisture content was measured and weight loss reached 34.89 ± 1.87% and 65.5 ± 1.23%, correspondingly. The results displayed that with the extension of withering time, the temperature and weight loss of the leaves showed a gradual increase and the rate of dehydration increased, demonstrating the achievement of results that are similar to those stated in earlier research [6,11]. This indicates that throughout the withering of white tea, the harvested tea leaves, especially after 12 h, gradually lose water, the temperature increases with time, and the leaves are continuously in a state of abiotic stress.

The quality control (QC) samples were prepared from a mixture of sample extracts and were used to analyze the reproducibility of the samples under the same processing method. During the instrumental analysis, one QC sample was inserted for every 10 samples tested in order to monitor the reproducibility of the analytical process. As shown in Figure S1, the total ion current (TIC, i.e., the intensity of all ions in the mass spectrometry at each time point, summed and continuously depicted) and the MRM metabolite detection multi-peak (ion flow spectrum of multiple substance extraction, XIC) were plotted for the mixed sample QC samples, with the retention time (RT) of the metabolite detection in the horizontal coordinate and the ion flow intensity (intensity in cps, count per second) in the vertical coordinate. The results showed a high curve overlap for the total ion flow of metabolite detection, i.e., consistent retention time and peak intensity, indicating that the mass spectrometer has good signal stability when detecting the same sample at different times. The high stability of the instrument provides an important guarantee for the reproducibility and reliability of the data. As shown in Figure S1, the total ions current (TIC, i.e., the intensity of all ions in the mass spectrometry at each time point is summed and continuously depicted) and the MRM metabolite detection multi-peak (ion flow spectrum of multiple substance extraction, XIC) were plotted for the mixed sample QC samples, with the retention time (RT) of the metabolite detection in the horizontal coordinate and the ion flow intensity (intensity in cps, count per second) in the vertical coordinate. The results showed a high curve overlap for the total ion flow of metabolite detection, i.e., consistent retention time and peak intensity, indicating that the mass spectrometer has good signal stability when detecting the same sample at different times. The high stability of the instrument provides an important guarantee for the reproducibility and reliability of the data.

Then, PCA analysis revealed the general change in nonvolatile compounds, including flavonoids and saccharides, during the withering processing. From 0 to 30 h, the score graph clearly demonstrated stepwise variations and obvious distinctions for nonvolatile substances, and metabolite alterations primarily occurred during the withering phase (Figure 1C), suggesting that the 12 h and 30 h withering phases are crucial steps for the transformation of flavonoid glycosides in white tea. Verified standards, MS2 spectra, and metabolomic datasets were used (Metlin and Human Metabolome database), and 75 nonvolatile chemicals were eventually selected, containing 13 saccharides, 46 flavonoid glycosides, and 16 flavonoid aglycones (Table S3).

### 3.2. Dynamic Changes in Nonvolatile Compounds (Flavonoids and Water-Soluble Saccharides) during the Withering Period

**Figure 2:**
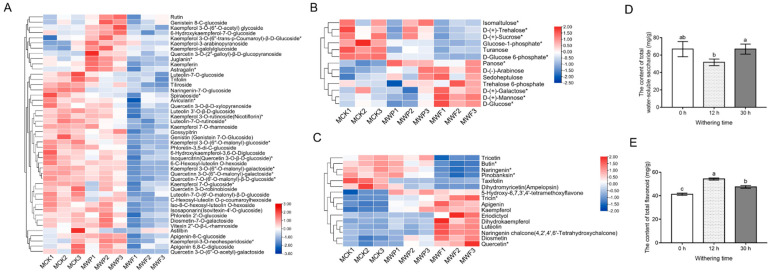
Heat map of the levels of differential nonvolatile compounds and content changes of flavonoids and water-soluble saccharides during the withering period. (**A**) Heat map of contents of flavonoid glycosides detected at different time periods. (**B**) Heat map of contents of water-soluble saccharides detected at different time periods. (**C**) Heat map of contents of flavonoid aglycones detected in different time periods. (**D**) The contents of total flavonoid at different time periods. (**E**) The contents of total water-soluble saccharides at different time periods. Each value represents the mean ± SD (n = 3). Values with different letters (a–c) differ from each other significantly (*p* < 0.05). Note: * the isomeric labeling indicates that these compounds were very similar in structure and co-flux will occur in mass spectrometry, which can be used for preliminary identification, but cannot be clearly distinguished. MCK, MWP, and MWF represent metabolomic samples with withering times of 0 h, 12 h, and 30 h, respectively.

Among the above 75 nonvolatile compounds, including flavonoids and water-soluble saccharides, all showed extremely significant changes (VIP ≥ 1 and absolute Log2FC ≥ 1) during the withering period (Figure 2A–C and Table S3). As shown in Figure 2E, the total flavonoid contents during withering ranged from 41.22 ± 0.95 mg/g to 54.21 ± 0.88 mg/g. The total flavonoid content increased rapidly and reached a peak during 0~12 h. After 30 h of withering, the total flavonoid content decreased. However, compared to fresh leaves, the total flavonoid content still increased significantly by 6.53 mg/g during the final period. The change in total water-soluble saccharide content displayed an opposite trend to that of the flavonoids. With increasing withering time, the water-soluble saccharide content tended to decrease and then increase. Between 0 and 12 h, the water-soluble saccharide content decreased significantly. At the end of withering, the water-soluble saccharide content increased significantly and reached a level comparable to that before withering, with no significant difference (Figure 2D).

Flavonoid glycosides are important astringent compounds in teas, and their hydrolyzed products have a great influence on the flavor formation of white tea. There was a total of four classes of flavonoid glycosides identified, including 12 flavone glycosides, 28 flavonol glycosides, 2 isoflavone glycosides, and 4 dihydroflavone glycosides. During the withering process of fresh leaves, the contents of most flavonoid glycosides showed a decreasing trend after 30 h (Figure 2A), which was consistent with a previously reported result [11]. The levels of most flavonoid glycosides decreased slightly or did not change significantly from 0 h to 12 h, while rutin, genistein 8-C-glucoside, kaempferol 3-O-(6″-O-acetyl) glycoside, kaempferol-3-arabinopyranoside, kaempferol-galolylglucoside, quercetin 3-O-(2″-galloyl)-β-D-glucopyranoside tended to increase significantly. Meanwhile, a total of five classes of flavonoid aglycones were detected, including two flavonols (quercetin, kaempferol), four dihydroflavonol (pinobanksin, dihydrokaempferol, taxifolin, ampelopsin), six flavones (apigenin, luteolin, diosmetin, tricetin, tricin, 5-hydroxy-6,7,3′,4′-tetramethoxyflavone), three dihydroflavone (naringenin, butin, eriodictyol), and one chalcone (naringenin chalcone). Among the flavonoid aglycones, the levels of tricetin, butin, naringenin, taxifolin, and dihydromyricetin significantly decreased after 30 h of withering, while the levels of 5-hydroxy-6,7,3′,4′-tetramethoxyflavone, tricin, kaempferol, apigenin eriodictyol dihydrokaempferol, luteolin, naringenin chalcone, quercetin, and diosmetin increased significantly. There were no significant differences in most of flavonoid glycosides in the early stages of withering processing. However, as withering processing continued, a large amount of water was lost from the tea leaves, cell membrane permeability increased, and the reactions of related proteases were enhanced. This led to a decrease in the content of flavonoid glycosides at the late stage of wilting and hydrolysis, forming glycosides and flavonoid aglycones.

Water-soluble saccharides confer the sweet and mellow taste to white tea infusions. All 13 water-soluble saccharides showed extremely significant changes in the withering step. The levels of six water-soluble saccharides, including D-(-)-arabinose, sedoheptulose, trehalose 6-phosphate, panose, and three hexose isomers, significantly increased after 30 h of withering, while the levels of two phosphorylated glucoses and four disaccharide isomers decreased significantly. Among them, two phosphorylated glucoses were involved in two major respiratory metabolic pathways: the pentose phosphate pathway and the glycolytic pathway, confirming the possible existence of strong respiratory effects of postharvest tea leaves during the withering process. The trend seen in trehalose 6-phosphate may be caused by the heat and drought stress on the leaves during the processing of white tea [23]. The increase in hexose isomers might be correlated with the metabolism of oligosaccharides and the degradation of flavonoid glycosides.

### 3.3. Proteomics Assessment throughout the Withering Phase

A vital phase in creating the white tea taste is the protracted withering time. To learn more about the optical process that causes alterations in flavonoid glycosides throughout withering, a TMT-labeling proteomics investigation was used to examine the variations in the after-harvest leaf proteome. Because of this, major alterations in metabolites and previous studies on glycosidic compounds metabolites have tended to use comparatively short withering or spreading times (<12 h) [24,25]. As a result, the proteomes of leaves that had been withering for 0 h, 12 h, and 30 h were studied.

**Figure 3:**
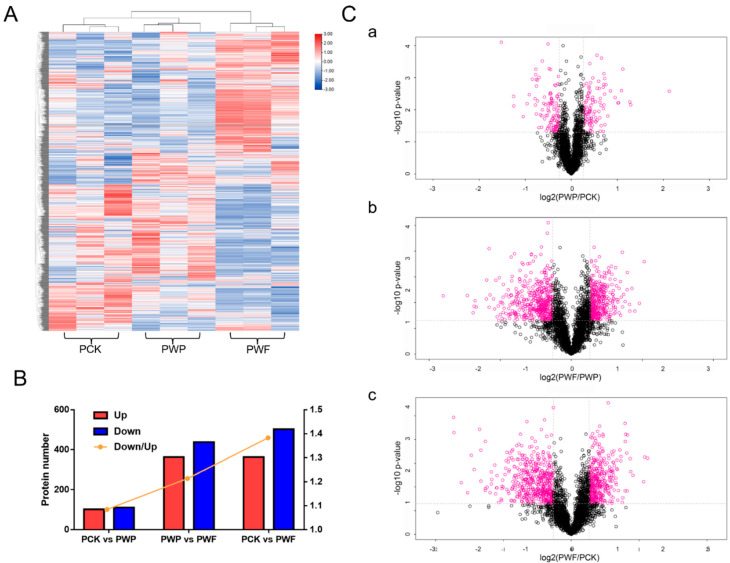
Overview of differentially expressed proteins (DPs). (**A**) Heat map of the types and contents of volatiles detected in different time periods. (**B**) Column chart showing DPs and the ratio of downregulated DPs to upregulated DPs (down/up). (**C**) The volcanic map was drawn by using the fold change in DPs between the two groups of samples (including a. PCK vs. PWP, b. PWP vs. PWF, c. PCK vs. PWF) and the *p*-value obtained by *t*-test, which was used to show the significant difference between the two groups. The abscissa is the change fold (logarithmic transformation with base 2), the ordinate is a significant *p*-value (logarithmic transformation with base 10), red dots represent proteins with significant difference (fold change was more than 1.2-fold and *p*-value < 0.05), black dots represent proteins with no difference. PCK, PWP, and PWF represent proteomic samples with withering times of 0 h, 12 h, and 30 h, respectively.

There were 14,646 peptides found (Figure S2A), with the majority of amino acids ranging from 5 to 21 (Figure S2B) in size; this shows that the sizes of the peptides were realistic. The mass inaccuracies of recognized peptides were mostly within 10 ppm (Figure S2C), showing strong peptide recognition reliability and accuracy. Furthermore, 73.7% of peptides had andromeda scores of over 20, with the median score of 31.29 (Figure S2D), showing that the andromeda values of MS2 spectra were quite good. Several proteins had molecular mass ranging from 10 to 80 kD (Figure S2E), showing that the molecular mass of the proteins discovered was realistic. Such peptides were combined to form 11,707 distinct proteins, 3224 of which were identified according to the *Camellia sinensis* genomic data [19]. The appearance of these proteins showed recognizable variation among different withering degrees (Figure 3A).

Fold modification values larger than 1.2 and *p* < 0.05 (from Student’ *t*-tests) were used to identify differentially expressed proteins (DPs). The quantity of DPs and the ratios of downregulated DPs to upregulated DPs increased with the withering duration, as shown in Figure 3B and Table S2, which is consistent with prior research [26]. The two factors, fold alteration and *p*-value acquired from Student’s *t*-tests, were used to plot the volcanoes together to indicate the significant differences between the data from the two groups (Figure 3C). This plot indicates that the protein changes were more dramatic during the time from 12 h to 30 h of white tea withering than from 0 h to 12 h.

As the time of withering increased, the intergroup differences in proteins became more apparent (Figure 3C), and the proteins content tended to be degraded (Figure 3B and Table S2). Combined with the clustering analysis of the expression patterns of all the differential proteins (Figure 3A), it was evident that protein expression was not consistent between the different sample groups, i.e., that there were differences in protein expression at three-time points in the withering process and differences in protein expression may lead to differences in the final metabolites.

### 3.4. Assessment of Differentially Expressed Proteins’ GO and KEGG Pathway Enrichment

For high-throughput proteomics research, understanding which functional or biological processes are harmed by the withering treatment is a priority. We used Blast2Go software to annotate all the recognized proteins in this project with GO function, and then performed GO function enrichment examination of DPs by Fisher’s exact test technique.

As displayed in Figure S3 and Table S4, the results of the analysis indicate that important biological procedures, for instance “negative regulation of nucleic acid-templated transcription”, “negative regulation of RNA biosynthetic process”, “positive regulation of transcription initiation from RNA polymerase II promoter”, “positive regulation of DNA-templated transcription initiation”, and “regulation of DNA-templated transcription initiation”, and molecular functions, including “TBP-class protein binding”, “proteasome-activating ATPase activity”, and “transcription factor binding”, and differential cellular components, such as “proteasome accessory complex”, “proteasome regulatory particle”, “chaperone complex and nuclear proteasome complex”, were significantly altered during the period of ongoing withering.

**Figure 4:**
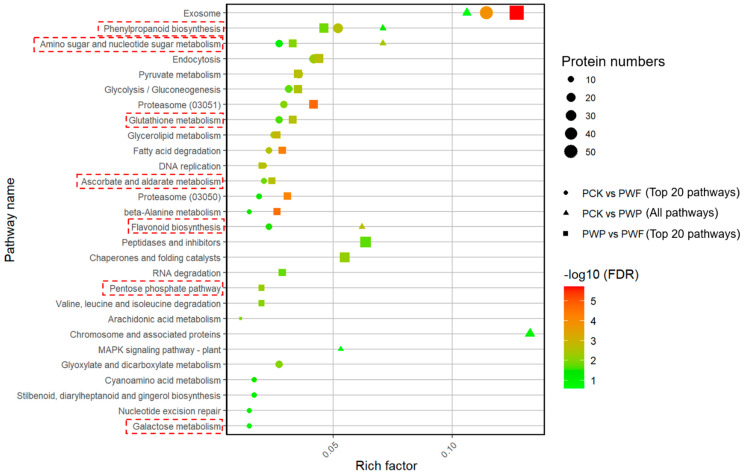
Functional enrichment analysis of differentially expressed proteins (DPs). The significantly enriched KEGG pathways of the DPs in threes group (including PCK vs. PWP, PWP vs. PWF, PCK vs. PWF). PCK, PWP, and PWF represent proteomic samples with withering times of 0 h, 12 h, and 30 h, respectively.

KEGG pathway enrichment assessment was used to better understand the internal relationships among DPs and the interaction between enriched DPs and the production of taste chemicals. As displayed in Figure 4 and Table S5A–C, specific pathways related to the biosynthesis and metabolism of flavonoids and water-soluble saccharides, such as “phenylpropanoid biosynthesis”, “flavonoid biosynthesis”, “pentose phosphate pathway”, “galactose metabolism”, and “amino sugar and nucleotide sugar metabolism”, were considerably enriched. Furthermore, numerous pathways associated with the oxidative stress and antioxidant defense in plants were also considerably enriched, such as “glutathione metabolism” and “ascorbate and aldarate metabolism”.

### 3.5. Expression Patterns of Key Proteins in Flavonoids Biosynthesis Pathways

**Figure 5:**
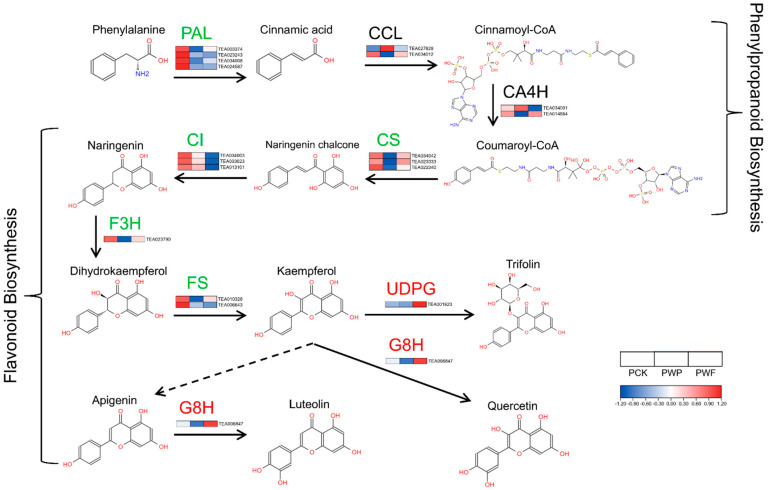
Effects of withering on protein expression in phenylpropanoid biosynthesis and flavonoid biosynthesis pathways. PAL: Phenylalanine ammonia-lyase; CCL: 4-coumaroyl CoA ligase; CA4H: Cinnamic acid 4-hydroxylas; CS: Chalcone synthase; CI: Chalcone isomerase; F3H: Flavanone 3-hydroxylase; FS: Flavonol synthase; UDPG: UDP-glycosyltransferas; G8H: Geraniol 8-hydroxylase. Note: green font indicates a downward trend, red font indicates an upward trend. PCK, PWP, and PWF represent proteomic samples with withering times of 0 h, 12 h, and 30 h, respectively.

Based on the changes observed in flavonoid glycosides and flavonoid aglycones during the withering process and the enrichment results of DPs, we focused on the expression patterns of key proteins related to flavonoids biosynthesis pathways. Figure 5 and Table S6C show the expression patterns of key proteins involved in phenylpropanoid biosynthesis and flavonoid biosynthesis pathways, respectively. As shown in Figure 5, the phenylalanine ammonia-lyases (PAL, TEA023243, TEA024587) involved in phenylpropanoid biosynthesis were downregulated during the withering process. The CSs changed with a pattern of obvious reduction, followed by a partial rebound, while the CIs decreased significantly in the last stages of withering. The trend of CIs was consistent with their associated metabolite, naringenin. However, the content of naringenin chalcones showed a significant increase after a long period of withering. This might be related to the hydrolysis of naringenin chalcone glycosides, such as naringenin chalcone 4′-O-glucoside, during the withering process. The CCLs and CA4Hs showed no significant trend in the phenylpropanoid biosynthesis pathways.

The F3Hs in flavonoid biosynthesis showed a significant decrease after 12 h of withering, then a partial increase. The content of dihydrokaempferol, the related metabolite of F3H, was significantly increased. The expression of FSs decreased significantly during the withering of white tea, but the kaempferol content increased significantly after 30 h of withering. G8H, which catalyzes the conversion of kaempferol and apigenin to luteolin and quercetin, respectively, showed a significant upregulation in white tea after withering. The trend in the content of flavonoid aglycones, including apigenin, luteolin, and quercetin, was also consistent with G8H. UDPG and CA3M in flavonoid glycoside biosynthesis were upregulated to varying degrees after withering, which suggested that the biosynthesis of flavonoid glycosides may persist until the final stage of withering. This is also consistent with the results of previous studies [11]. However, the change trend in UDPG expression was contrary to the trend occurring in the levels of trifolin.

To sum up the above, we found that the trends in the content of flavonoid glycosides and flavonoid aglycones did not match those of their corresponding proteases in the flavonoid biosynthesis pathways. So we speculated that the increases in major flavonoid aglycones, such as kaempferol, quercetin, apigenin, and luteolin, were possibly related to the degradation of flavonoid glycosides during the withering process. In addition, coupled with the fact that flavonoids are chemically stable, the degradation of flavonoid glycosides may, to some degree, occur simultaneously with biosynthesis, although no studies on this have been conducted.

### 3.6. Characterization of Flavonoid Glycoside Degradation-Related Co-Expressed Gene Networks and Major Candidate Proteins

**Figure 6:**
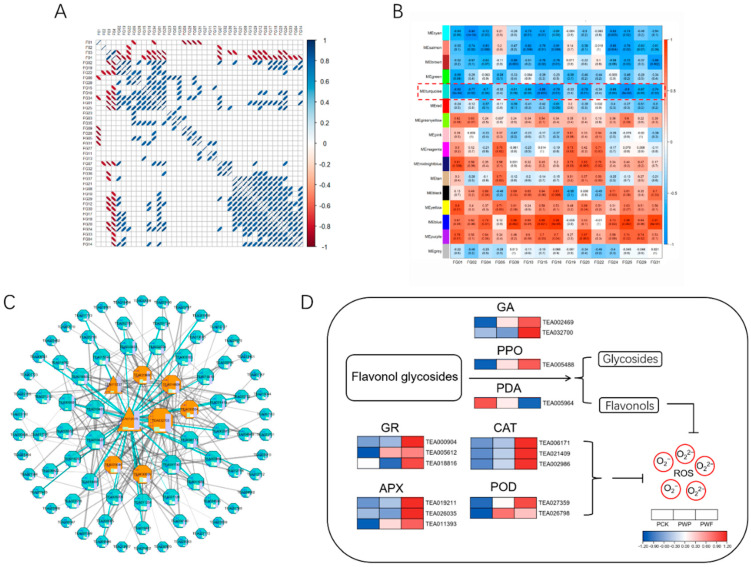
Co-expression modules’ identification based on weighted gene co-expression network analysis (WGCNA) and the protein change in the degradation of flavonol glycosides to mitigate oxidative stress. (**A**) Pearson’s correlation coefficient (PCC) between every two metabolites (*p* < 0.05). (**B**) Module–trait associations based on Pearson correlations. Each row represents one module, and each column corresponds to one screened flavonol glycoside. (**C**) Protein co-expression network for the MEturquoise module. The top 200 proteins based on topological overlap measure (TOM) values were used to construct the co-expression network and were visualized by Cytoscape 3.8.2 software. The triangles represent the transcription factors, orange octagons represent the key hub proteins. (**D**) The expression patterns of key proteins in the degradation of flavonol glycosides. FG01: Apigenin-8-C-glucoside; FG02: Iso-8-C-hexosyl-luteolin O-hexoside; FG03: 6-C-Hexosyl-luteolin O-hexoside; FG04: C-Hexosyl-luteolin O-p-coumaroylhexoside; FG05: Kaempferol-3-arabinopyranoside; FG06: Kaempferol 7-O-rhamnoside; FG07: Quercetin 3-O-β-D-xylopyranoside; FG08: Astragalin; FG09: Trifolin; FG10: Kaempferol 7-O-glucoside; FG11: 6-Hydroxykaempferol-7-O-glucoside;FG12: Isoquercitrin; FG13: Kaempferol 3-O-(6″-O-acetyl) glycoside; FG14: Quercetin 3-O-(6′′-O-acetyl)-galactoside; FG15: Kaempferol 3-O-(6″-O-malonyl)-galactoside; FG16: Kaempferol 3-O-(6′′-O-malonyl)-glucoside; FG17: Quercetin-7-O-(6′-O-malonyl)-β-D-glucoside; FG18: Quercetinn 3-O-(6′′-O-malonyl)-galactoside; FG19: Kaempferol 3-O-(6″-trans-p-Coumaroyl)-β-D-Glucoside; FG20: Kaempferol-3-O-neohesperidosid; FG21: Kaempferol 3-O-rutinoside(Nicotiflorin); FG22: Kaempferol-galolylglucoside; FG23: Quercetin 3-O-(2″-galloyl)-β-D-glucopyranoside; FG24: 6-Hydroxykaempferol-3,6-O-Diglucoside; FG25: Luteolin 3′-O-β-D-glucoside; FG26: Luteolin-7-O-glucoside; FG27: Luteolin-7-O-(6′-O-malonyl)-β-D-glucoside; FG28: Luteolin-7-O-rutinoside; FG29: Apigenin 6,8-C-diglucoside; FG30: Rutin; FG31:Tiliroside; FG32: Spiraeoside; FG33: Genistin; FG34: Juglanin; FG35: Kaempferin; FG36: Avicularin; FG37: Bioquercetin; F01: Quercetin; F02: Kaempferol; F03: Apigenin; F04: Luteolin; GA: Glycosidase; PPO: Polyphenoloxidase; PDA: Primeverosidase; GR: Glutathione reductase; CAT: Catalase; APX: Ascorbate peroxidase; POD: Peroxidase; ROS: Reactive oxygen species. PCK, PWP, and PWF represent proteomic samples with withering times of 0 h, 12 h and 30 h, respectively.

The relationships between metabolic contents may be used to show the links between metabolites, which can aid in the improvement in metabolic networks and discovering new metabolic pathways. Therefore, to further explore the relationship between the major flavonoids aglycones in flavonoid biosynthesis pathways and the degradation of flavonoid glycosides during the withering of white tea, PCC was determined between the relative contents of four flavonoid aglycones (including kaempferol, quercetin, apigenin, and luteolin) and 37 flavonoid glycosides with molecular structures corresponding to theirs. In Figure 6A, 15 flavonoid glycosides, which included C-hexosyl-luteolin O-p-coumaroylhexoside, apigenin-8-C-glucoside, iso-8-C-hexosyl-luteolin O-hexoside, kaempferol-3-arabinopyranoside, trifolin, kaempferol 7-O-glucosdie, kaempferol 3-O-(6′′-O-malonyl)-galactoside, kaempferol 3-O-(6′′-O-malonyl)-glucoside, kaempferol 3-O-(6′′-trans-p-Coumaroyl)-β-D-Glucoside, kaempferol-3-O-neohesperidoside, kaempferol-galolylglucoside, 6-hydroxykaempferol-3,6-O-diglucoside, luteolin 3’-O-β-D-glucoside, apigenin 6,8-C-diglucoside, and tiliroside, showed significant negative correlations (*p* < 0.05) with the related flavonoids aglycones.

To examine the protein controlling network of the degradation of flavonoid glycosides during withering, WGCNA was used to find co-expressed protein sets. The integrated dynamic assessment of WGCNA discovered 15 co-expression modules (labeled and emphasized in various colors), each of which had 40 to 865 proteins (Figure S3 and Table S7). Using the module–trait relationship to study the contents of 15 major flavonoid glycosides (listed above) considered, phenotypic traits showed that the turquoise module had the most substantial negative association with the accumulation of most flavonoid glycosides (Figure 6B). The proteins in the turquoise module were used for further analyses.

In order to investigate the functional qualities of the turquoise module, a KEGG enrichment examination was carried out. As shown in Figure S5, the KEGG pathways “pyruvate metabolism”, “glycolysis/Gluconeogenesis”, and 2 oxidative stress-related pathways (including “ascorbate and aldarate metabolism”, “peroxisome”), were enriched in the turquoise module.

Based on the top 200 topological overlap measure (TOM) values, the 83 proteins in the turquoise module were used to create the co-expression networks, and between them, the transcription factors (TFs) and proteins associated with flavonoid glycoside degradation were designated as vital hub proteins. As displayed in Figure 6C and Table S6C, a total of eight key hub proteins were recognized in the turquoise module, containing two transcription factors, HY5 TF (TEA012075) and TCP13 TF (TEA015233), one cytosolic beta-endo-N-acetyglucosaminidase (ENGase, TEA032700), and one aconitate hydratase (TEA033504), one 26S proteasome regulatory subunit (TEA014859), one alanine aminotransferase (TEA023090), one anthocyanidin reductase (TEA030009). Among them, ENGase was certified to cleave the O-glycosidic connection between the two GlcNAc remains of the N-glycan core structure in arabidopsis thaliana [27], and its relative expression increased substantially at 30 h of withering. This suggests that the ENGase also has the potential to cleave the O-glycosidic of flavonoid glycosides in tea leaves. Furthermore, in this module, the expressions of the other crucial hub proteins also increased significantly in the late stage of withering. The outcomes of these analyses showed that the upregulation of key hub proteins might be accountable for the degradation of flavonoid glycosides in tea throughout the withering procedure. Their roles in the degradation of flavonoid glycosides throughout the withering procedure must be further examined.

Based on the co-expression networks of the turquoise module, it may be deduced that these important hub proteins are not only related to a large number of edge proteins but also are strongly related to one another, suggesting that they cooperate or compete in the breakdown of flavonoid glycosides in the form of a protein network. Furthermore, coupled with the results of the KEGG enrichment analysis in the module, the degradation of flavonoid glycosides may be the outcome of a mixture of factors involving oxidative stress, saccharides metabolism, and hydrolysis by glycosidases.

### 3.7. Degradation of Flavonol Glycosides to Mitigate Oxidative Stress

The withering process of white tea is a process of fresh leaves being harvested and then experiencing drought, heat stress, and gradual aging. While tea leaves undergo these stresses, they are often accompanied by oxidative stress. Among the flavonoid glycosides of tea, flavonol glycosides are an important component [9]. In addition, studies have shown that flavonol aglycones, the hydrolysis products of flavonol glycosides, such as kaempferol and quercetin [28,29], have high antioxidant activity, and there is a strong possibility that catabolism of flavonol glycosides to aglycones is a required mechanism for antioxidant action in plants [30,31]. Based on these views, we focused on the changes in expression of relevant antioxidant and glycoside-degrading enzymes during the withering process of tea leaves. As shown in Figure 6D, the protein expressions of glutathione reductases (GRs), catalases (CATs), ascorbate peroxidases (APXs), and peroxidase (PODs) increased gradually with the withering process of white tea, which also indicated the gradual accumulation of reactive oxygen species (ROS) in the cells of tea leaves. The relative expression of beta-glucosidase 12 (TEA002469) and polyphenol oxidase (TEA005488) showed a significant upward trend in white tea at the late stage of wilting, consistent with the trend in ENGase. These results, which are consistent with the results of previous studies [15], combined with the above-mentioned change patterns of flavonoid glycoside substances, indicate that the degradation of flavonoid glycosides is very strong in the late stage of withering of white tea. In contrast, the trend in primeverosidase (TEA005964) expression decreased gradually with time, suggesting that the protein family of primeverosidase may not be the main enzyme category related to flavonol glycoside hydrolysis in the withering processing of tea.

## 4. Conclusions

In summary, our study combined nonvolatile metabolomics and proteomics to analyze the postharvest leaves of white tea and revealed the effects of withering on flavonoid glycosides metabolism. After a long period of withering (12–30 h), the harvested tea leaves will be in an extreme state affected by multiple abiotic stresses, including drought, heat stress, or mechanical damage. When the tea leaves experience these adversities, the cellular oxidative stress balance gradually break down. The biosynthesis and degradation of flavonoid glycosides may be a protection mechanism of antioxidants in tea leaves, which may also be the mechanism by which white tea changes the content and components of flavonoid glycosides in its leaf cells through the withering process to form a special flavor. We identified a co-expression module network that was highly correlated with the degradation of flavonoid glycosides during the withering process. The analysis of the proteins in the module revealed that different categories of glycosidases may be involved in the hydrolysis of flavonol glycosides during the withering process of white tea, and the metabolism of flavonol glycosides may have some interactions with saccharides’ metabolism. Therefore, these proteins in the module can be regarded as key candidate proteins worthy of further investigation.

## Data Availability

All of the data included in this study are available upon request by contacting the corresponding author.

## Supplementary Materials

The following supporting information can be downloaded at: https://www.mdpi.com/article/10.3390/foods11091226/s1, Figure S1: Quality control (QC) sample mass spectrometry detection TIC overlay; Figure S2: Proteomic assay quality analysis during the withering period of white tea; Figure S3: Top 20 significantly enriched gene ontology (GO) of the DPs in PCK vs. PWP vs. PWF; Figure S4: Clustering dendrograms of genes and module division; Figure S5: Significantly enriched KEGG pathways of the DPs in the MEturquiose; Table S1: Grouped list of metabolic and proteomic samples; Table S2: Numbers of differentially expressed proteins during the withering period; Table S3: Nonvolatile components identified and dynamic changes in contents (LC-MS intensity) during white tea processing; Table S4: Significantly enriched gene ontology (GO) terms during the withering process; Table S5: Significantly enriched KEGG pathways during the withering process; Table S6: Part of differentially expressed proteins and their expression abundance during the withering period; Table S7. All proteins KME.
