# Supplementary material for: Metabolomics Combined with Proteomics Provide a Novel Interpretation of the Changes in Flavonoid Glycosides during White Tea Processing
Source: Foods. 2022 Apr 24;11(9):1226. doi: 10.3390/foods11091226 (PMC9103810; doi:10.3390/foods11091226)
Supplement: Supplementary file 1 [file foods-11-01226-s001.zip › Figure S1.pdf]

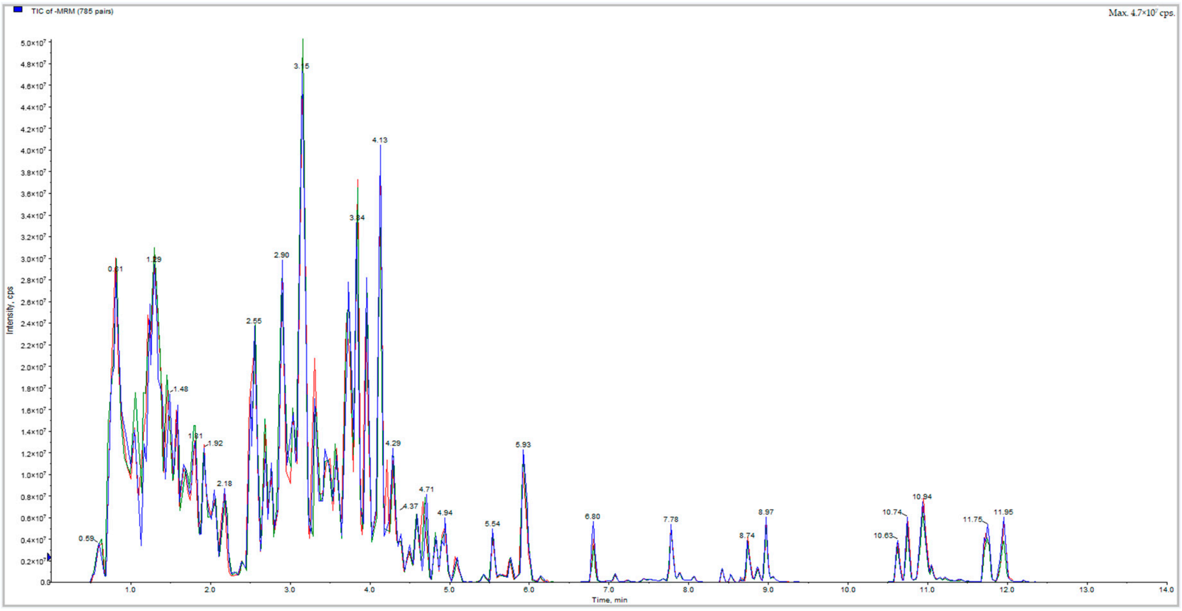

(A)

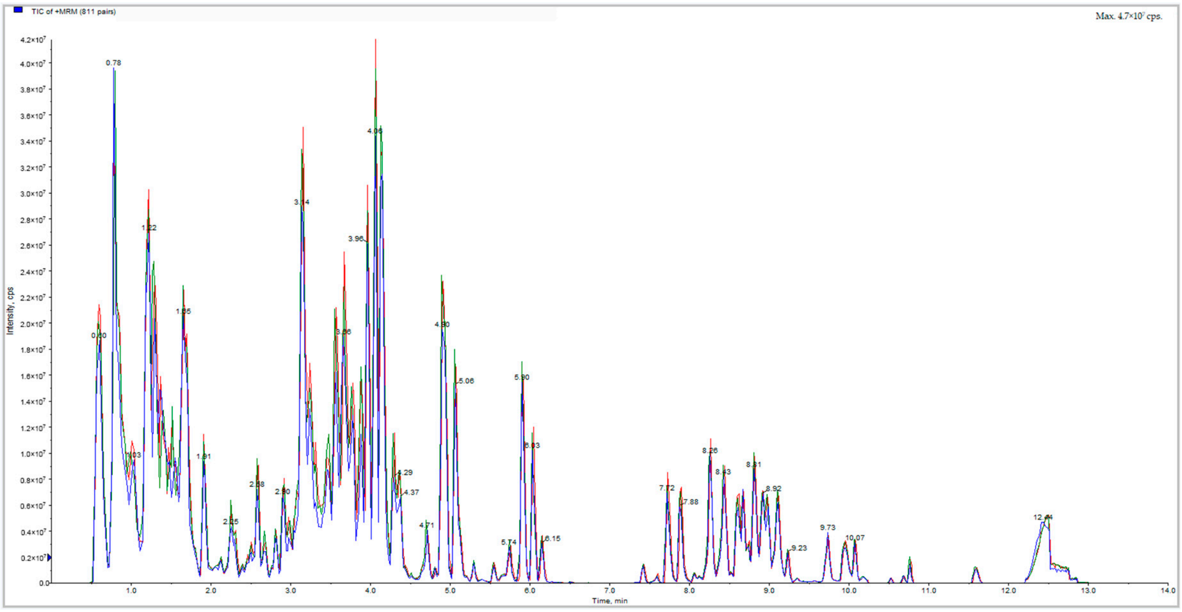

(B)

**Figure S1.** The quality control (QC) sample mass spectrometry detection TIC overlay. (A) QC MS tic overlap-N of the tea sample; (B) QC MS tic overlap-P of the tea sample.
