# Supplementary material for: Metabolomics Combined with Proteomics Provide a Novel Interpretation of the Changes in Flavonoid Glycosides during White Tea Processing
Source: Foods. 2022 Apr 24;11(9):1226. doi: 10.3390/foods11091226 (PMC9103810; doi:10.3390/foods11091226)
Supplement: Supplementary file 1 [file foods-11-01226-s001.zip › Figures S2-S5.pdf]

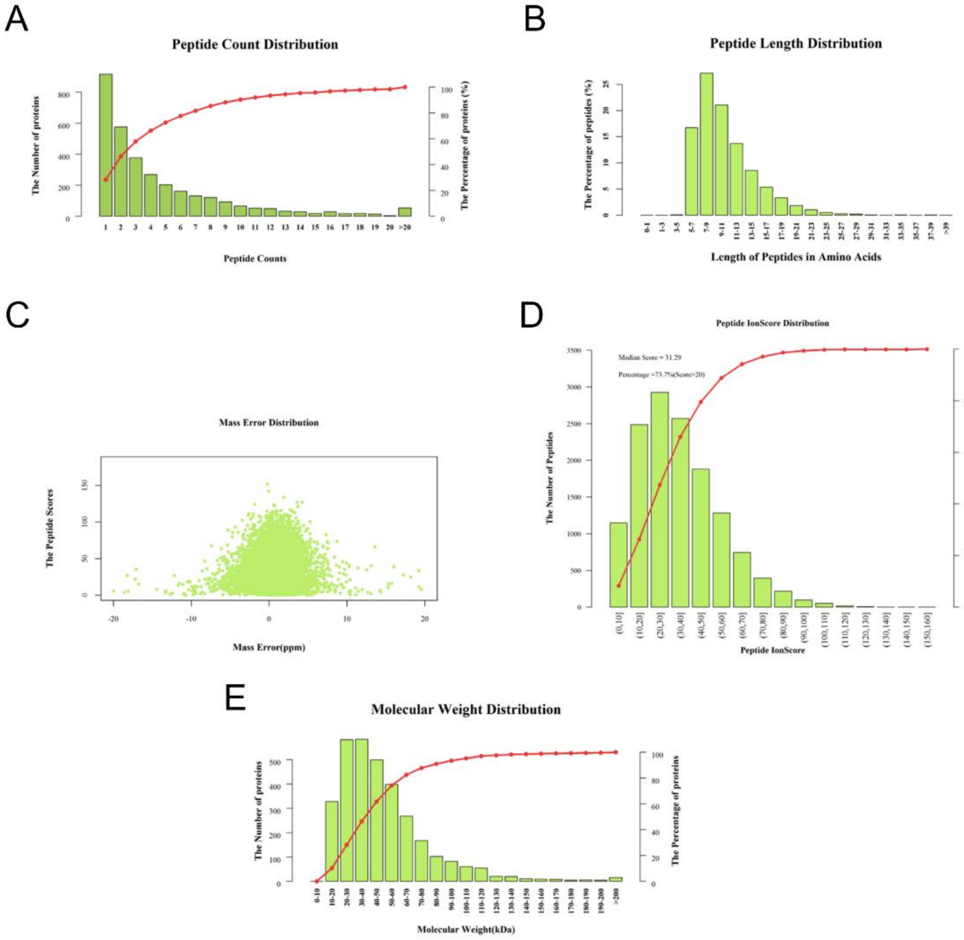


**Figure S2.** Proteomic assay quality analysis during the withering period of white tea. The distributions of peptide number (A) and length (B); (C) The mass error distribution of identified peptides; (D) The andromeda score distribution of identified peptides; (E) The distributions of protein molecular weight.

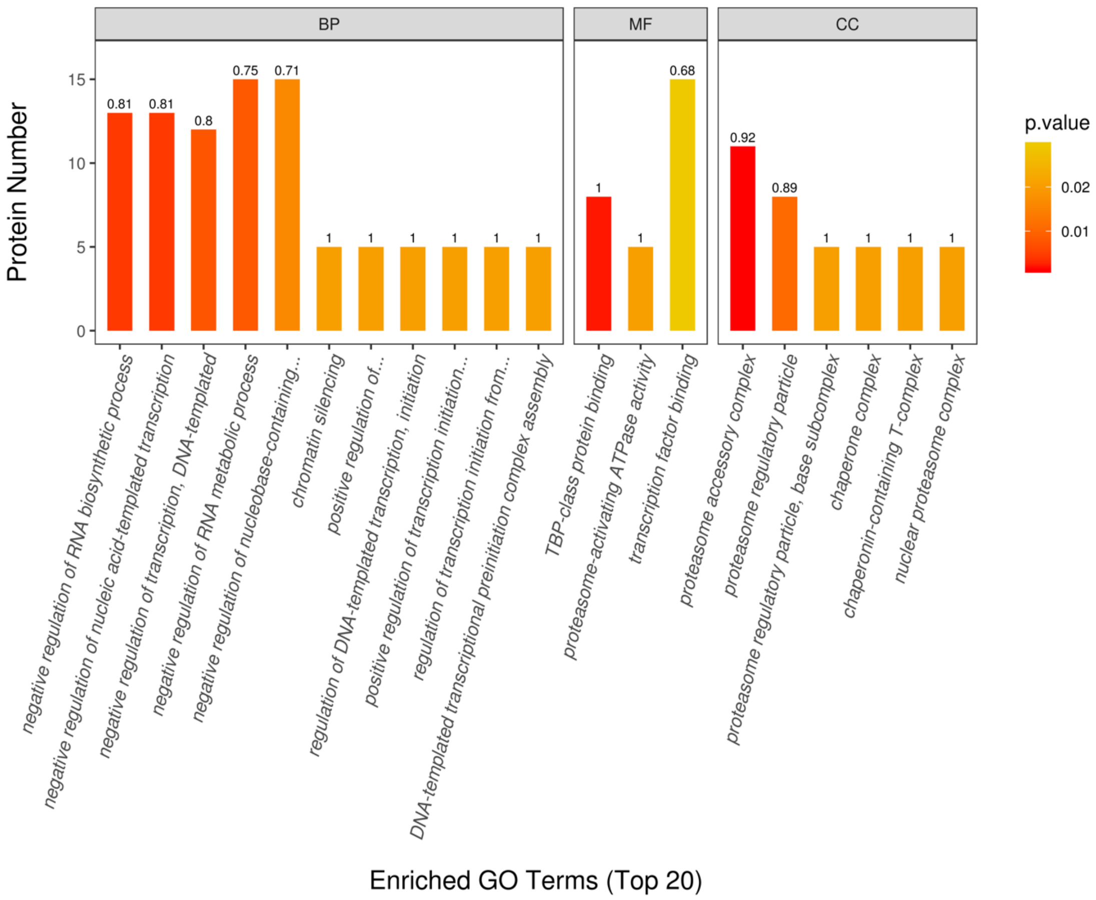


**Figure S3.** Top 20 significantly enriched gene ontology (GO) of the DPs in PCK vs PWP vs PWF.

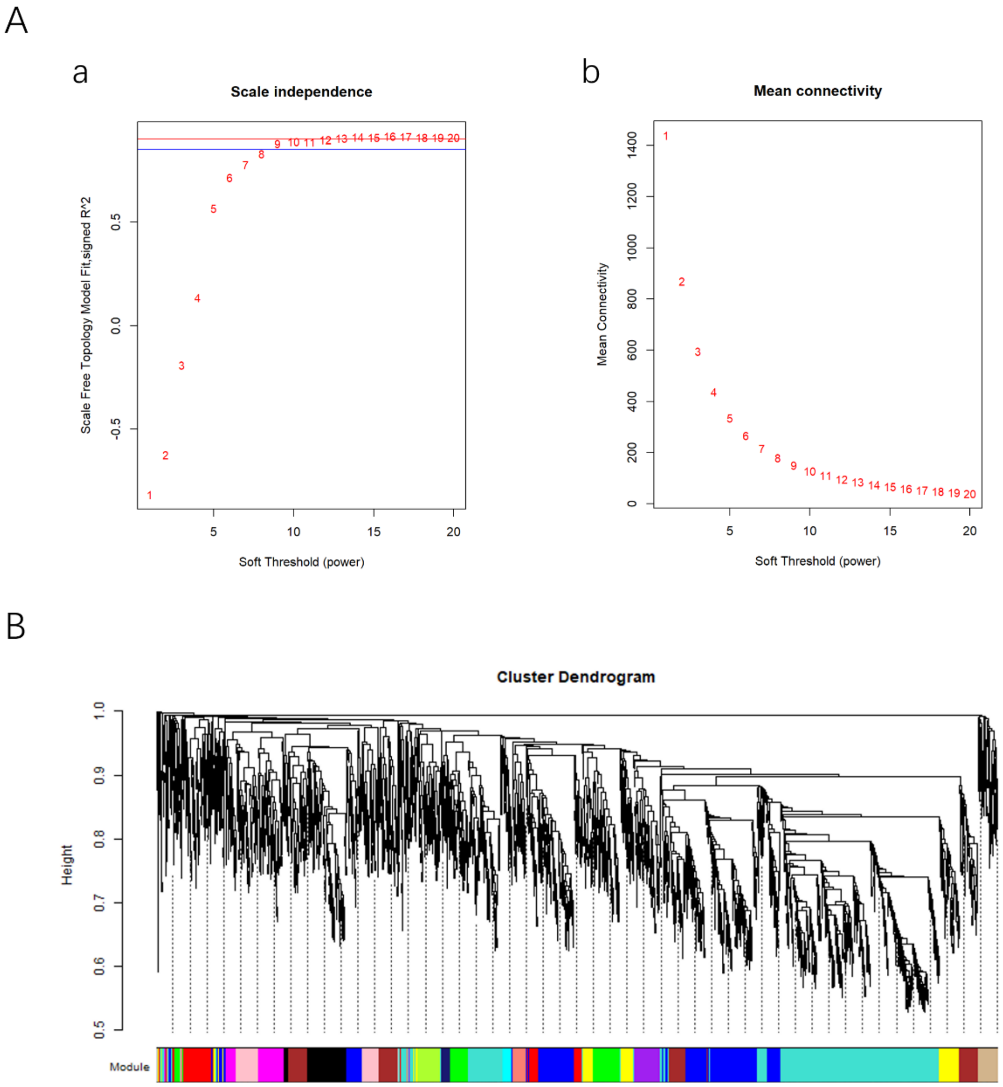


**Figure S4.** Clustering dendrograms of genes and module division. (A) Analysis of network topology for various soft-thresholding powers. The scale-free fit index (y-axis) as a function of the soft-thresholding power (x-axis) (a), and the mean connectivity (degree, y-axis) as a function of the soft-thresholding power (x-axis) (b). (B) Protein clustering on TOM-based dissimilarity, module division by dynamic tree cut and different colors represent different modules.

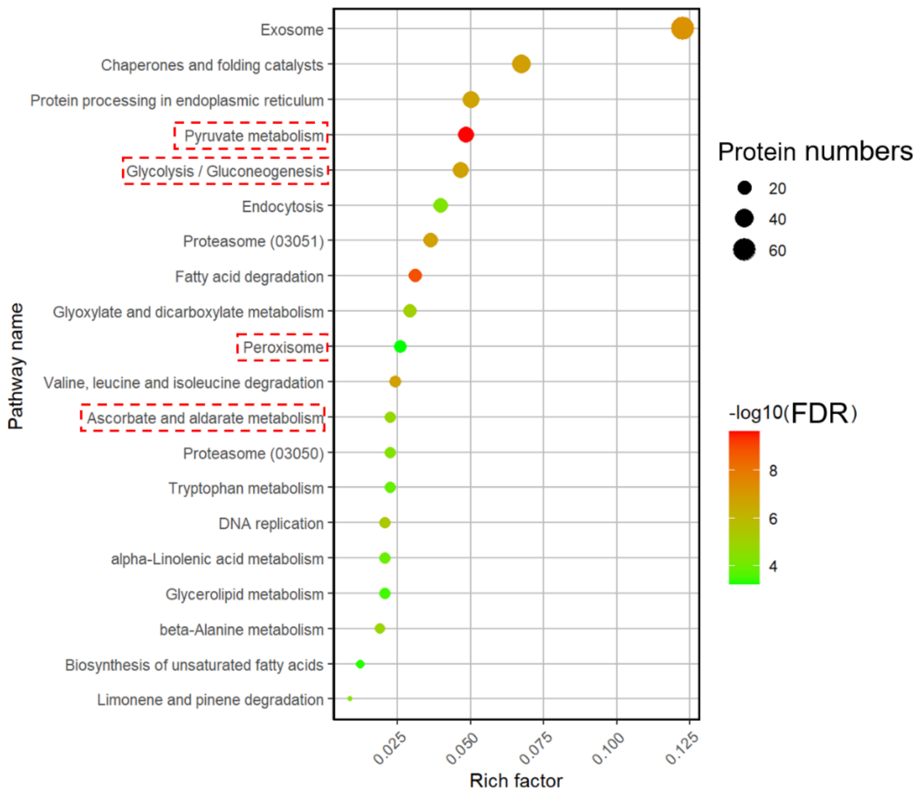


**Figure S5.** The significantly enriched KEGG pathways of the DPs in the MEturquiose
