# Supplementary material for: Metabolomics Combined with Proteomics Provide a Novel Interpretation of the Changes in Flavonoid Glycosides during White Tea Processing
Source: Foods. 2022 Apr 24;11(9):1226. doi: 10.3390/foods11091226 (PMC9103810; doi:10.3390/foods11091226)
Supplement: Supplementary file 1 [file foods-11-01226-s001.zip › Table S1 and S2.pdf]

Table S1. The grouped list of metabolic and proteomic samples

Table S1-A. Metabolic samples

Withering time/h	Group of metabolomics sample	Three biological replicates
0	MCK	MCK1<br>MCK2<br>MCK3
12	MWP	MWP1<br>MWP2<br>MWP3
30	MWF	MWF1<br>MWF2<br>MWF3

Table S1-B. Proteomics samples

Withering time/h	Group of proteomics sample	Three biological replicates
0	PCK	PCK1<br>PCK2<br>PCK3
12	PWP	PWP1<br>PWP2<br>PWP3
30	PWF	PWF1<br>PWF2<br>PWF3

Table S2. Numbers of differentially expressed proteins during the withering period

Comparisons	Up	Down	Down/Up	All
PCK vs PWP	102	110	1.08	212
PWP vs PWF	362	438	1.21	800
PCK vs PWF	363	502	1.38	865

Comparisons: differential comparison groups; Up: up-regulation of differentially expressed proteins; Down: down-regulation of differentially expressed proteins; All: all differentially expressed proteins.
